# From glioma gloom to immune bloom: unveiling novel immunotherapeutic paradigms-a review

**DOI:** 10.1186/s13046-024-02973-5

**Published:** 2024-02-12

**Authors:** Moksada Regmi, Yingjie Wang, Weihai Liu, Yuwei Dai, Shikun Liu, Ke Ma, Guozhong Lin, Jun Yang, Hongyi Liu, Jian Wu, Chenlong Yang

**Affiliations:** 1 Department of Neurosurgery, Peking University Third Hospital, Peking University, Beijing, 100191 China; 2 Center for Precision Neurosurgery and Oncology of Peking University Health Science Center, Peking University, Beijing, 100191 China; 3 Peking University Health Science Center, Beijing, 100191 China; 4 Henan Academy of Innovations in Medical Science (AIMS), Zhengzhou, 450003 China; 5 National Engineering Research Center for Ophthalmology, Beijing, 100730 China; 6 Engineering Research Center of Ophthalmic Equipment and Materials, Ministry of Education, Beijing, 100730 China; 7 Beijing Key Laboratory of Ophthalmology and Visual Sciences, Beijing, 100730 China

**Keywords:** Gliomas, Immune checkpoints, Inhibitors, Immunotherapy

## Abstract

In tumor therapeutics, the transition from conventional cytotoxic drugs to targeted molecular therapies, such as those targeting receptor tyrosine kinases, has been pivotal. Despite this progress, the clinical outcomes have remained modest, with glioblastoma patients' median survival stagnating at less than 15 months. This underscores the urgent need for more specialized treatment strategies. Our review delves into the progression toward immunomodulation in glioma treatment. We dissect critical discoveries in immunotherapy, such as spotlighting the instrumental role of tumor-associated macrophages, which account for approximately half of the immune cells in the glioma microenvironment, and myeloid-derived suppressor cells. The complex interplay between tumor cells and the immune microenvironment has been explored, revealing novel therapeutic targets. The uniqueness of our review is its exhaustive approach, synthesizing current research to elucidate the intricate roles of various molecules and receptors within the glioma microenvironment. This comprehensive synthesis not only maps the current landscape but also provides a blueprint for refining immunotherapy for glioma, signifying a paradigm shift toward leveraging immune mechanisms for improved patient prognosis.

**Supplementary Information:**

The online version contains supplementary material available at 10.1186/s13046-024-02973-5.

## Main text

The evolution of immunotherapy since its inception in 1985 has revolutionized the field of oncology, adding an entirely new approach to cancer treatment. The landscape of cancer immunotherapy is diverse, encompassing a range of strategies like immune checkpoint inhibitors (ICIs), adoptive cell transfer therapy, tumor-specific vaccines, and targeted small molecule inhibitors.

**Fig. 1:**
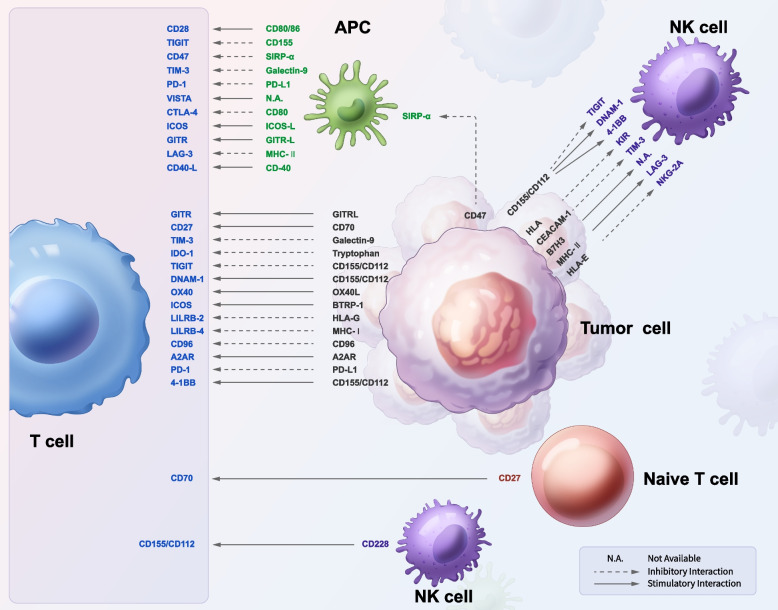
Immunoregulatory checkpoints in the tumor microenvironment. Illustration of immunoregulatory checkpoints in the tumor microenvironment, detailing cell interactions. T cells, with receptors like PD-1 and CTLA-4, mainly receive inhibitory signals from APC and tumor cell ligands such as PD-L1. Conversely, receptors like CD28 on T cells interact with ligands like CD80/86 on APCs, initiating activation. NK cells are controlled by activating receptors such as 4-1BB and inhibitory receptors like NKG 2A, which interact with HLA-E on tumor cells. Tumor cells themselves express ligands like PD-L1 to inhibit T-cell function and CD47 to evade immune clearance. Naive T cells show CD27 as a primary activation marker. Arrows indicate the direction of interaction between ligands and their corresponding receptors: solid for stimulation and dashed for inhibition

**Fig. 2:**
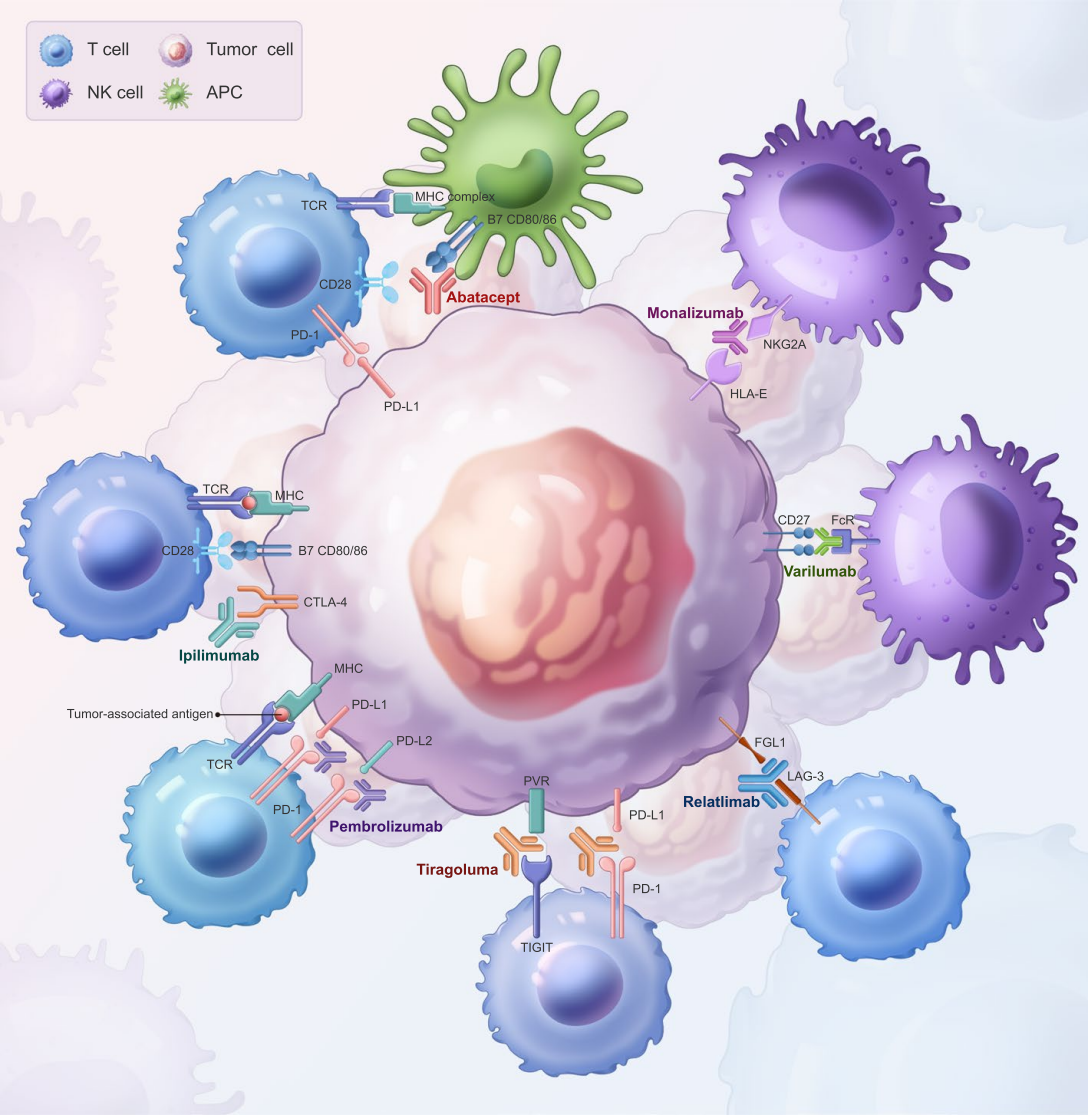
Mechanisms of action of certain immunotherapeutic drugs. Schematic representation of the mechanisms of action of various immunotherapeutic drugs in the tumor microenvironment. Drugs intervene on T cells, APCs, and NK cells ligands and receptors to modulate immune responses against tumor cells. Abatacept blocks the interaction between CD28 on T cells and CD80/86 on APCs, inhibiting T cell activation. Ipilimumab targets CTLA-4 on T cells, preventing it from competing with CD28 for CD80/86 on APCs, thereby promoting T cell activation. Pembrolizumab binds to PD-1 on T cells, preventing its interaction with PD-L1 and PD-L2 on tumor cells, which would otherwise lead to T cell inhibition. Tiragolumab targets TIGIT on T cells, blocking its interaction with PVR on tumor cells, and similarly preventing T cell inhibition. Monalizumab binds to NKG2A on NK cells, blocking its interaction with HLA-E on tumor cells, which enhances NK cell-mediated cytotoxicity. Varlilumab facilitates attack on tumor cells through CD27. Relatlimab inhibits LAG-3 on T cells, preventing it from binding to FGL1 on tumor cells, thus averting T cell exhaustion

At the heart of these immune responses are immune checkpoints (Fig. 1), which are crucial for modulating the intensity and duration of the immune system's action. These checkpoints play dual roles: they maintain self-tolerance to prevent autoimmunity, while also modulating immune responses against external pathogens or malignancies [1]. Cancer cells have developed sophisticated mechanisms to manipulate the immune system's regulatory network, particularly through the amplification of immune checkpoint pathways like the programmed cell death protein-1 (PD-1) / programmed death ligand-1 (PD-L1) and cytotoxic T lymphocyte-associated protein 4 (CTLA-4). This manipulation effectively suppresses the immune system's ability to mount a robust antitumor response. ICIs counteract this by targeting an array of coinhibitory and costimulatory receptors (Fig. 2), along with their ligands, to enhance antitumor immunity.

ICIs hold the potential to reshape cancer treatment. Notably, the use of anti-PD-1 monoclonal antibodies has led to improved survival rates across various cancer types, including hepatocellular carcinoma, non-small cell lung cancer (NSCLC), renal cell carcinoma (RCC), melanoma, urothelial carcinoma, and a range of other solid tumors [2–6]. Moreover, anti-CTLA-4 agents have exhibited survival benefits in patients with metastatic melanoma and are presently under clinical evaluation for other malignancies, including NSCLC, RCC, and prostate cancer [7–9].

**Fig. 3:**
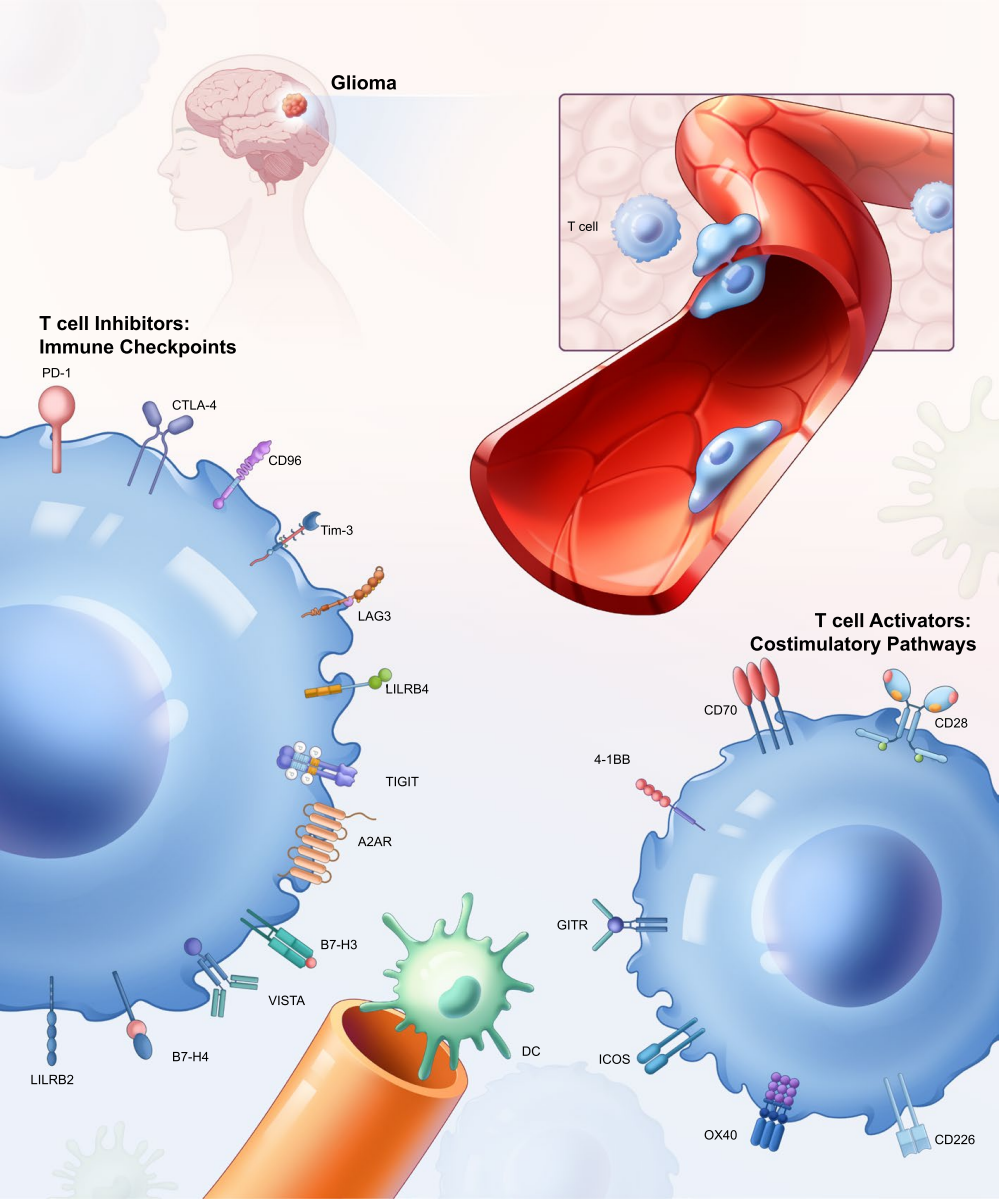
T cells in the glioma microenvironment. Diagrammatic sketch of T cells and their modulatory elements within the glioma microenvironment. On the left, key inhibitory receptors on T cells such as PD-1, CTLA-4, LAG3, TIM-3, TIGIT, CD96, A2AR, VISTA, LILRB2, LILRB4, B7-H3, and B7-H4 are highlighted. These receptors attenuate T cell effector functions to prevent autoimmunity and maintain self-tolerance. In the glioma microenvironment, APCs and tumor cells often exploit these inhibitory pathways to evade immune detection. On the right, T cell activators including CD28, CD226, OX40, ICOS, GITR, 4-1BB, and CD70 are depicted. These activators engage with their respective ligands on APCs and tumor cells, enhancing T cell activation, proliferation, and survival, thereby promoting an anti-tumor immune response

Gliomas pose a significant challenge in oncology [10]. In the glioma microenvironment, immune cells aggregate to combat tumor progression, with T cells being a crucial component of this response (Fig. 3). However, the brain's immune environment, including the blood–brain barrier, restricts the entry of immune cells and therapeutics. Furthermore, gliomas foster an immunosuppressive tumor microenvironment (TME), characterized by significant inter- and intratumoral heterogeneity and cellular plasticity. These characteristics contribute to immune evasion and pose significant hurdles to effective immunotherapy. Moreover, the immunobiology of gliomas differs from that of other tumors, necessitating tailored approaches for successful treatment. Given the frequent recurrence after traditional treatments such as surgery, radiation, and chemotherapy, and considering glioma's distinct pathogenic traits, the exploration of ICIs in its treatment has gained critical importance.

However, recent studies in 2018 have shown that only 43.6% of cancer patients are eligible for immunotherapy, with an anticipated response rate of 12.46%, with substantial disparities across different cancer types [11]. While highlighting these complexities, we will in the following sections discuss the various receptors and their associated potential treatments.

### T-Cell activators: costimulatory pathways

In gliomas, a unique expression profile of costimulatory molecules differentiates them from other cancers. Only six molecules—HHLA2, TNFRSF14, TNFRSF18, TNFRSF25, TNFRSF6B, and TNFSF9—are underexpressed in glioma tumors, while 48 others exhibit increased expression [12]. This differential expression significantly influences patient prognosis and therapeutic response, suggesting unique therapeutic opportunities. Additionally, enrichment analyses revealed distinct correlations between costimulatory molecules and immunotherapy prediction pathways, underscoring their unique involvement in glioma biology [12].

#### CD28

CD28 provides a crucial second signal alongside T-cell receptor (TCR) ligation for naive T-cell activation. The unique epigenetic, transcriptional, and post-translational changes in T cells triggered by CD28 ligation cannot be achieved by TCR ligation alone, making it a significant target for therapeutic modulation in glioma treatment [13–15].

Recent studies have targeted the CD28/CTLA-4 cosignaling pathway to ameliorate transplant rejection and autoimmune diseases [13, 15]. CD28 blockers abatacept and belatacept have been developed as targeted therapies for transplant rejection and autoimmune disease relative to calcineurin inhibitors and antiproliferative agents (ICIs used in clinical trials for non-glioma cancer immunotherapies are summarized in Supplementary Table S1). However, the efficacy of CD28 blockade is only still being evaluated in clinical trials and its efficacy in the brain has TME still not been verified [15]. Also, immunotherapeutic approaches for GBM treatment reveal pronounced T-cell dysfunction, exemplified by increased CD8+CD28- T cells, characterized by limited TCR diversity and impaired activation [16]. This dysfunction, exacerbated by the glioma TME, underscores the potential of targeting the CD28 pathway to augment glioma immunogenicity. Therefore, strategies to mitigate the immunosuppressive milieu and revitalize T-cell activation, including by modulating CD8+CD28- T cell pathways, could significantly enhance immunotherapy efficacy in GBM patients.

#### CD27

CD70, present on antigen-activated T cells and in tumors like meningioma and GBM, can induce lymphocyte apoptosis, T-cell exhaustion, and immune escape, and modulate immune suppression via macrophages [17–24].

Modulating the CD70-CD27 interaction is emerging as a promising approach for solid tumor therapy and targeting leukemia stem cells [25]. Unique in its quiescent state during homeostasis, CD70 presents as a cancer-specific target [17]. CD27 agonists, such as varlilumab, have shown potential in enhancing antitumor responses and synergizing with ICIs [26]. It has also been reported that CD70, via a receptor-dependent pathway, induces T-cell apoptosis and promotes tumor migration [27]. These agents also enhance chimeric antigen receptor T (CAR-T) cell therapy efficacy, although blocking the CD27-CD70 interaction can diminish this effect [28].

In vivo studies on murine glioma models indicate that CD70-targeted therapies can cause tumor regression, but contrasting evidence suggests that the CD27 pathway might also suppress T lymphocytes. CD70 expression in glioma cells can trigger T-cell apoptosis and tumor growth inhibition, possibly due to NK cell activity or differences in the apoptosis-regulating Siva pathway. Additionally, CD70 overexpression in tumors attracts immunosuppressive macrophages [29, 30]. Recent studies have introduced HERA-CD27L, a hexavalent CD27 agonist, which significantly enhances T-cell activation and induces antitumor immunity. This innovation in CD27 agonism, by creating a hexavalent structure, optimizes the binding and stimulation of T cells, thereby amplifying T-cell responses [31]. In both in vitro and in vivo studies, HERA-CD27L has been demonstrated to be effective at enhancing human T-cell activation post-stimulation and significantly boosting antigen-specific CD8+ T-cell responses [31]. These findings suggest that targeting the CD27 pathway with such agonists could be a promising strategy for enhancing immune response against gliomas. These findings collectively highlight the complex role of the CD27/CD70 pathway in glioma treatment.

#### 4-1BB

4-1BB (CD137) is an inducible receptor-like protein, whose expression is limited to activated T cells post-TCR triggering, rather than being present in naive or resting CD4+ and CD8+ T cells [32]. Simultaneously, 4-1BBLs undergo transient induction on activated professional antigen-presenting cells (APCs), responding to signals from CD40s and tumor-associated macrophages (TAMs) [33]. The interaction between 4-1BB and 4-1BBL, or the administration of agonist monoclonal antibodies, has a cosignaling effect. This shields antigen-specific cytotoxic T lymphocytes from apoptosis, fosters cytokine production, and stimulates T-cell proliferation [34, 35]. Consequently, 4-1BB agonists wield potent antitumor effects, making them popular in immunotherapy for various advanced cancers.

Interestingly, studies have reported synergistic survival benefits when 4-1BB agonists are combined with PD-1 blockade or radiotherapy [36]. Notably, the efficacy of single-agent 4-1BB agonists is limited by dose-dependent toxicity. Hence, current clinical research predominantly centers on combination therapies aimed at mitigating undesirable effects. One innovative approach engineered proteins capable of concurrently targeting 4-1BB and tumor stroma or tumor antigens, effectively overcoming the potential side effects of 4-1BB agonists and yielding remarkable tumor regression in murine models [37]. In parallel, bispecific immunotherapy agents that simultaneously and complementarily target PD-L1 blockade and conditional 4-1BB stimulation within a single molecule have been explored. This pioneering treatment strategy has demonstrated promising results in terms of manageable safety and disease control, particularly in patients who have exhibited resistance to prior PD-1 immunotherapy [38]. Thus, the potential of combined therapies holds significant promise in various cancer contexts, and with urelumab and utomilumab already showing fairly good tolerance with antitumor activities, strategies targeting 4-1BB specific to GBM warrant further research.

#### GITR

The glucocorticoid-induced tumor necrosis factor receptor (GITR) and its ligand, GITRL, represent a critical immunoregulatory axis within the TNF superfamily. GITR is basally expressed on CD4+ and CD8+ T cells, with expression upregulated post-activation [39]. GITRL, predominantly expressed on APCs, also increases upon activation. This axis, in conjunction with TCR stimulation, activates naive T cells, promoting their proliferation and secretion of key cytokines, including IL-2 and IFN-γ [40–42].

Agonistic monoclonal antibodies targeting GITR enhance cancer immunotherapy efficacy, as evidenced by their efficacy in preclinical tumor models and modulation of Treg populations [43]. These antibodies are particularly potent against advanced solid tumors [44–46] and gliomas, and exhibit heightened Treg activity [47]. Recent studies have highlighted the benefits of combining local GITR agonist injections with systemic therapy, significantly improving survival rates [47, 48]. Additionally, the synergistic effect of these antibodies with stereotactic radiosurgery in murine models leads to increased survival, effector cell infiltration, and cytokine production [49].

Preclinical findings have catalyzed clinical trials of GITR agonists TRX518 and BMS-986156. BMS-986156 demonstrated a favorable safety profile when used alone and in combination with nivolumab, whereas TRX518 elicited limited clinical response [46]. However, GBM remains a relatively untapped frontier in the exploration of GITR agonists, thus warranting further investigations.

#### ICOS

Inducible T-cell co-stimulator (ICOS), is a costimulatory molecule involved in T-cell activation, that is predominantly expressed on activated CD4+ T cells and interacts with ICOSL on APCs [50–52]. This interaction is crucial for T follicular helper cell differentiation, enhancing high-affinity antibody production and humoral immunity [50, 53].

In glioblastoma, elevated ICOS levels within TMEs, compared to peripheral blood, indicate its prognostic value [30]. However, ICOS's association with T regulatory cells (Tregs) is dichotomous because of the role of Tregs in dampening antitumor immunity [50]. This complexity highlights ICOS as a therapeutic target in glioblastoma.

Recent studies reveal ICOSL expression in myeloid populations within mesenchymal glioblastoma subtypes, affecting both tumor and stromal cells [50]. Increased ICOS expression post-treatment potentially indicates immunotherapy responsiveness in glioblastoma [51]. Current clinical trials exploring ICOS targeting through agonistic and antagonistic antibodies, including vopratelimab and feladilimab, have shown that ICOS is safe but are pending efficacy results.

Adoptive immunotherapy research, particularly CAR-T cells engineered against ICOS and EGFRvIII, shows promise in vitro against glioma cells (U87) and in xenograft mouse models, marked by IFN-γ secretion and tumor growth inhibition [54, 55]. These advances suggest the viability of ICOS-targeted therapies in glioblastoma treatment, despite ongoing debates over the clinical relevance of the U87 tumor model.

#### OX40

The burgeoning interest in OX40 as a target for cancer immunotherapy is underscored by its demonstrated efficacy in combination treatments [56–58]. The absence of severe adverse events associated with OX40 agonists posited them as viable addition to existing treatment regimens [59]. OX40, when paired with agents such as anti-CTLA-4, significantly enhances T-cell mediated antitumor responses, suggesting a synergistic potential that extends beyond monotherapy applications and correlates with improved GBM patient prognoses, with high OX40L mRNA levels in GBM linked to longer progression-free survival [57, 60–63].

OX40 agonists used alone, such as MEDI6383 and GSK3174998, have shown efficacy in multiple cancers, including pancreatic, lung, breast cancers, and NSCLC [64–68]. The synergistic effect of OX40 monoclonal antibodies with vaccines has demonstrated reduced tumor growth, increased apoptosis, and T-lymphocyte revitalization [63, 69]. Further, a triple combination therapy (vaccine, anti-PD-1 antibody, and OX40 agonist) in glioma murine models yielded strong Th1 responses and enhanced survival [70]. In melanoma, particularly phosphatase and tensin homolog-null variants, OX40 activation has been pivotal in augmenting the cytotoxic function of CD8+ T cells, a finding that could reshape approaches for treating immune-resistant tumors [71]. The ongoing development of novel anti-OX40 antibodies and their characterization for immune activation echo a dynamic landscape of therapeutic innovation, with huge potential for enhancing immunotherapy efficacy in glioma patients. These developments also corroborate the necessity for a paradigm shift toward more integrated, combination-based approaches in cancer immunotherapy.

### T-cell inhibitors: immune checkpoints

Single-cell analyses have revealed the heterogeneity of the glioma microenvironment, including the distinct roles of myeloid cells, glioma cells, and T cells, suggesting that interactions mediated by each checkpoint are crucial for understanding and improving immunotherapeutic strategies for glioma treatment [72].

#### PD-1

PD-1/PD-L1 inhibition is a promising strategy in cancer therapy, particularly beneficial in high-grade cases where PD-L1 correlates with poor prognosis [73, 74]. Ongoing trials continue to assess the efficacy of these inhibitors [73]. However, in the context of malignant gliomas, anti-PD-1 therapy has achieved limited clinical success, attributed to the complex glioma microenvironment. Glioma-associated microglia/macrophages (GAMs) play a pivotal role in this setting. While M1-like GAMs support the therapeutic response, the predominance of M2-like GAMs fosters resistance [75]. Notably, PD-L1 ablation modifies the M2-like phenotype of GAMs, thereby potentiating anti-PD-1 therapy [75]. Furthermore, gliomas impair the antigen-presenting capacity of GAMs, thereby diminishing the antitumor immunity of CD4+ T cells in the context of PD-1 blockade.

Nevertheless, PD-1/PD-L1 inhibitors remain the most extensively studied in clinical trials for GBM treatment (ICIs in clinical trials for glioma immunotherapy are summarized in Table 1) [76–78]. For instance, the efficacy of the anti-PD-1 antibody nivolumab in treating gliomas has been extensively studied. The CheckMate 143, a phase III randomized clinical trial, did not meet the primary endpoint for median overall survival (mOS) in patients with recurrent glioblastoma (rGBM) compared with bevacizumab. However, a post-subgroup analysis suggested potential benefits from nivolumab, particularly in patients with methylated MGMT promoters. Neoadjuvant PD-1 blockade in GBM may enhance antitumor immune responses [79, 80].

**Table 1:** Overview of ICIs in clinical trials for glioma immunotherapy

Target	Intervention	Author	Year	Journal	Country	Clinical Trial Phase	Enrolment	Comments
**PD-1**	Pembrolizumab	Sahebjam et al	2018	*Neuro Oncol*	US	I	32	ClinicalTrials.gov ID NCT03426891. Combined with vorinostat and temozolomide. No dose-limiting adverse events were observed, thrombocytopenia and fatigue were the most common adverse events
		Migliorini et al	2019	*Neuro Oncol*	CH	I/II	24	ClinicalTrials.gov ID NCT03665545. The IMA950/poly-ICLC vaccine was safe and well tolerated, median overall survival was 19 months for glioblastoma patients
		-	-	-	US	II	35	Unpublished: ClinicalTrials.gov ID NCT02852655. Better survival with pre-and post-operative treatment; upregulation of T-cell and interferon-γ-related gene expression
		Groot et al	2020	*Neuro Oncol*	US	II	15	ClinicalTrials.gov ID NCT02337686. Indirect signs of immune engagement were observed; anti-PD-1 monotherapy was seen as insufficient for the majority of glioblastoma patients
		-	-	*-*	US	N.A	12	Unpublished: ClinicalTrials.gov ID NCT02658279. For patients with recurrent malignant glioma with a hypermutator phenotype
		Iwamoto et al	2022	*Neuro Oncol*	US	II	60	ClinicalTrials.gov ID NCT03661723. Re-irradiation with pembrolizumab was overall well tolerated and achieved comparable efficacy to traditional methods
	Cemiplimab	Reardon et al	2020	*Neuro Oncol*	US	I/II	52	ClinicalTrials.gov ID NCT03491683. Delivered by electroporation in combination with INO-5401 and INO-9012, overall survival was found encouraging
	Nivolumab	-	-	*-*	US	II	37	Unpublished: ClinicalTrials.gov ID NCT03557359. For recurrent or progressive IDH mutant gliomas
		-	-	*-*	US	II	94	Unpublished: ClinicalTrials.gov ID NCT03743662. With radiation therapy and bevacizumab for recurrent MGMT-methylated glioblastoma
		-	-	*-*	US	II	95	Unpublished: ClinicalTrials.gov ID NCT03718767. For IDH-mutant gliomas with and without hypermutator phenotype
		Omuro et al	2023	*Neuro Oncol*	Multinational	III	550	ClinicalTrials.gov ID NCT02617589. Safety confirmed but no survival advantage demonstrated in unmethylated MGMT promotor GBM
		Lim et al	2022	*Neuro Oncol*	UK	III	693	ClinicalTrials.gov ID NCT02667587. No improvement in progression-free survival; overall survival data is pending
		Reardon et al	2020	*JAMA Oncol*	Multinational	III	369	ClinicalTrials.gov ID NCT02017717. No difference in overall outcome vs bevacizumab; Median OS 9.8 months with nivolumab
		Schalper et al	2019	*Nat Med*	ES	II	30	ClinicalTrials.gov ID NCT02550249. Increased chemokine transcripts, immune cell infiltration, and T-cell receptor clonal diversity were observed post-surgery
		-	-	*-*	US	I	60	Unpublished: ClinicalTrials.gov ID NCT04606316. In combination with ipilimumab and surgery
**PD-L1**	Atezolizumab	Weathers et al	2020	*J Clin Oncol*	US	I/II	60	Unpublished: ClinicalTrials.gov ID NCT03174197. In combination with temozolomide and radiation Therapy
		Tiu et al	2021	*Neuro Oncol*	UK	I/II	51	ClinicalTrials.gov ID NCT03673787. With ipilimumab and short-course radiotherapy in MGMT unmethylated GBM. Preliminary evidence of antitumor activity
	Retifanlimab	Campian et al	2022	*J Clin Oncol*	US	II	55	ClinicalTrials.gov ID NCT03532295. Retifanlimab combined with radiotherapy and bevacizumab in patients with glioma was well-tolerated and had encouraging OS and PFS
	Avelumab	Neyns et al	2019	*J Clin Oncol*	BE	II	52	ClinicalTrials.gov ID NCT03291314. Was well tolerated when used in combination with axitinib, but did not meet the threshold for activity justifying further investigation
		Jacques et al	2021	*Neurooncol Adv*	CA	II	30	Unpublished: ClinicalTrials.gov ID NCT03047473. No apparent improvement in overall survival was used for newly diagnosed glioblastoma patients
	Bintrafusp alfa	-	-	*-*	Multinational	I	105	Unpublished: ClinicalTrials.gov ID NCT02517398. The same trial explores intervention efficacy for other cancer types, and favorable results have been published
	Durvalumab	-	-	*-*	US	II	36	Unpublished: ClinicalTrials.gov ID NCT02794883. Durvalumab used with tremelimumab or alone
		Reardon et al	2019	*J Clin Oncol*	US, AU	II	84	ClinicalTrials.gov ID NCT02336165. Was well tolerated when combined with radiotherapy and seemed to have efficacy among patients with new unmethylated glioblastoma
**CTLA-4**	Ipilimumab	Sloan et al	2018	*J Clin Oncol*	US	I	32	ClinicalTrials.gov ID NCT02311920. Usage was found safe and tolerable
		-	-	*-*	US	II	37	Unpublished: ClinicalTrials.gov ID NCT04145115
		-	-	*-*	US	II/III	485	Unpublished: ClinicalTrials.gov ID NCT04396860
	Tremelimumab	-	-	*-*	US	II	36	Unpublished: ClinicalTrials.gov ID NCT02794883. Tremelimumab used with durvalumab or alone
**LAG-3** **4-1BB**	BMS 986016 Urelumab	Lim et al	2020	*J Clin Oncol*	US	I	63	ClinicalTrials.gov ID NCT02658981. The maximum tolerated dose has been identified
**TIM-3**	MBG453 Spartalizumab	-	-	*-*	US	I	15	Unpublished: ClinicalTrials.gov ID NCT03961971

 Furthermore, multidrug combinations targeting PD-1, PD-L1, and CTLA-4 have been found to be effective in GBM mouse models [81]. Studies have shown that the expression of PD-L1 and the presence of exhausted tumor-infiltrating lymphocytes (TILs) expressing multiple immune checkpoints, including PD-1, are indicative of an adaptive resistance mechanism to anti-PD-1/PD-L1 therapy [82–84]. Also, the combination of temozolomide with anti-PD-1 antibodies has demonstrated survival improvements in glioma models, but concurrent dexamethasone treatment may negatively impact this approach [85]. Studies are even combining these inhibitors with immunomodulatory agents like Toll-like receptor agonists and cytokines [86]. Investigations into PD-L1 expression regulation in glioma cells suggest the potential for improved outcomes through combinations with IFN-γ and other cytokines [87]. This underscores the necessity for a multifaceted approach in immunotherapy, potentially involving combination therapies that target multiple aspects of the TME to overcome resistance mechanisms.

#### CTLA-4

In glioma, CTLA-4 expression is significantly correlated with both the WHO grade and isocitrate dehydrogenase (IDH) status, indicating its varied roles in tumor biology and patient prognosis. It plays a critical role in modulating T-cell activation through its interaction with CD80 and CD86 on APCs. Its upregulation in gliomas correlates with poor prognosis, especially in patients with high-grade variants, indicating its viability as a therapeutic target in glioblastoma [88].

Anti-CTLA-4 therapies, designed to block CTLA-4 synthesis and thereby boost T cell activity, have shown variable effectiveness in glioma models [88–90]. Notably, combining anti-CTLA-4 agents with other treatments, such as focal radiation and 4-1BB activation, or anti-PD1 therapy, has improved outcomes in preclinical settings [90, 91].

Current research is investigating both agonistic and antagonistic CTLA-4 antibodies (e.g., ipilimumab and tremelimumab), aiming to refine CTLA-4 blockade strategies [92]. However, the associated risk of adverse effects, heightened in combination therapies, necessitates careful patient monitoring.

Deletion of CTLA-4 results in lymphocyte proliferation, confirming its role in early immune regulation [93]. Its similarity to CD28, but opposing function, highlights the B7/CTLA-4 checkpoint as a critical target for tumor-specific T-cell activation [93]. The CTLA-4 inhibitor ipilimumab, effective in treating malignant melanoma, including patients with brain metastases, underscores the need for further clinical exploration to determine its full therapeutic potential.

#### CD96

In glioma, CD96 is a significant immune checkpoint with a unique role in modulating the TME. Its expression, particularly elevated in high-grade, IDH-wildtype, and mesenchymal-molecular subtype gliomas, is strongly correlated with immune functions. Gene ontology analyses revealed a positive correlation between CD96 and immune-related genes, underscoring its potential influence on immune cell infiltration, including CD8+ T cells, Tregs, and macrophages [94]​​​​. CD96 also has differential immunomodulatory effects, and acts as an immunosuppressive agent in gliomas, revealing heightened expression in malignant phenotypes, with an adverse impact on overall survival, in contrast to its role in other cancers like melanoma where it is involved in activating immune responses​​. Furthermore, CD96 shows strong concordance with other immune checkpoint proteins such as TIGIT, CD226, and CRTAM, as well as established markers including PD-L1, CTLA-4, TIM-3, and STAT3, suggesting its potential for synergistic antitumoral effects in glioma immunotherapy​​ [95, 96].

Moreover, the potential of CD96 extends beyond its standalone impact, as it is believed to synergize positively with other checkpoint members, enhancing its significance in orchestrating immune responses [95]. This positions CD96 as a prospective checkpoint target, offering potential avenues for developing drugs that can complement existing immune checkpoint blockade therapies [95].

#### TIM-3

In gliomas, T cell immunoglobulin and mucin domain-containing protein 3 (TIM-3) works against the inflammatory response and inhibits T-cell-mediated immunity, which is critical for tumor defense, thus the expression of TIM-3 on T cells and various immune cells has been correlated with poor prognosis across various tumor types [97]. In glioblastoma, TIM-3 plays pivotal roles in myeloid cell responses with spartalizumab and other drugs, identifying the most functionally impaired CD8+ T-cell subset [98]. Extensive transcriptomic studies link TIM-3 to the mesenchymal molecular subtype in glioma [99]. TIM-3 expression and O-6-methylguanine-DNA methyltransferase promoter methylation in glioblastoma indicate an adverse prognosis [100]. Studies have shown that TIM-3 is abundantly expressed in glioblastoma and IDH-wild-type glioma, indicating its significance in these malignancies. Furthermore, in mouse models, TIM-3 has been found to affect the expression of immune-related molecules such as iNOS and PD-L1, highlighting its unique response to brain tumors and its significant role in intracellular and intercellular immunoregulation within the brain TME [101]. Preclinical studies have shown remarkable effectiveness in simultaneously targeting both the TIM-3 and PD-1 pathways, and ongoing trials predominantly focus on the same [102].

#### LAG-3

In glioma, LAG-3 is expressed on a subset of TILs, particularly in IDH-wildtype gliomas, and is associated with a more active inflammatory milieu characterized by higher TIL density. This expression pattern suggests a nuanced role for LAG-3 in the immune contexture of gliomas, potentially contributing to immune evasion mechanisms employed by tumor cells. The glioma microenvironment is influenced by the interaction of LAG-3 with various cellular components, including Tregs and dendritic cells (DCs). Tregs in the glioma microenvironment suppress other T cell populations through mechanisms involving direct cell contact or cytokine secretion, while DCs in gliomas often exhibit limited costimulatory molecule expression and favor Treg development. These interactions underscore the potential of LAG-3 as a target in glioma immunotherapy [103–105].

When combined with anti-PD-1, anti-LAG-3 therapies demonstrate exciting efficacy, particularly in overcoming PD-1 resistance [103]. Along with relatlimab, LAG-3 inhibitor fianlimab has shown encouraging results in bolstering cytotoxic T-cell-mediated tumor cell lysis [106]. Beyond MHC II, LAG-3 also engages with FGL1, α-synuclein fibrils, and lectins like galectin-3 and lymph nodes [107]. This interplay notably facilitates tumor immune escape by inhibiting the activation of antigen-specific T cells [105]. Yet, recent research has shown that it is the binding of LAG-3 to MHC II, not FGL1, that chiefly mediates T-cell suppression [105].

In glioma, LAG-3 is a potential marker for the mesenchymal molecular subtype, according to the Cancer Genome Atlas transcriptional classification [106], and warrants further exploration for potential clinical application.

#### LILRB4

LILRB4, a member of the leukocyte immunoglobulin-like receptor family, predominantly expressed on TAMs in gliomas and other cancers, exerts a significant immunosuppressive role within the TME and correlates with immune inhibitory receptors. Blockade of LILRB4 enhances tumor immune infiltrates, rebalances effector to regulatory T cell ratios, and modulates TAM phenotypes toward a less suppressive state, promoting the transformation of CD4+ T cells into Th1 effectors and CD8+ T cells into less exhausted states [108]. Studies using LILRB4-/- mice and anti-LILRB4 antibody treatments confirm its pivotal role, reducing tumor burdens and improving survival rates.

IO-202, an antibody targeting LILRB4, is advancing in phase I cohort expansion clinical trials for acute myeloid leukemia and chronic myelomonocytic leukemia, underscoring its clinical potential. Functionally, LILRB4 also plays a crucial role in tumor metastasis by orchestrating myeloid-derived suppressor cells (MDSCs) and inhibiting miR-1 family miRNAs, further emphasizing its significance in disease progression [109]. LILRB4 is a promising immunotherapeutic target for solid tumors and various diseases, warranting comprehensive exploration of its clinical applications.

#### TIGIT

In gliomas, the expression of immune checkpoint gene TIGIT (T-cell immunoreceptor with Ig and ITIM domains) is significantly elevated, correlating with advanced disease stages. This heightened expression suggests a potential role in tumor progression and aggressiveness, underscoring TIGIT's involvement in the immunosuppressive microenvironment typical of high-grade gliomas​[110]. Gene set enrichment analyses have further elucidated the biological signaling pathways of TIGIT, highlighting its contribution to immune suppression within this unique tumor context ​​[111]. Additionally, TIGIT promotes NK cell-dependent tumor immunity, as demonstrated in diverse mouse models [91, 112, 113].

TIGIT's ligands, CD155 and CD112, are expressed on tumor cells and APCs, further establishing its critical role in tumor immune responses [112]. Notably, TIGIT has demonstrated the capacity to dampen T-cell activation and proliferation, and TIGIT blockade has exhibited encouraging outcomes, resulting in enhanced antitumor immunity and prolonged survival, particularly in glioblastoma [114].

A notable advancement in this domain is the combination of the PD-L1 inhibitor atezolizumab with tiragolumab, an anti-TIGIT antibody. This tandem approach has exhibited early clinical activity, boasting an overall response rate of 46% in cases of advanced solid tumors [115]. Other interventions including etigilimab and vibostolimab are also under clinical trials and have thus far shown favorable results. However, the full scope of TIGIT's efficacy, whether as a standalone therapy or in combination with other immunotherapies, warrants further comprehensive investigation.

#### A2AR

Adenosine A1 and A2A receptors, abundant in the TME due to factors like hypoxia, cellular turnover, and enzymatic activity, mediate immunosuppression through A2A receptor signaling on various immune cells [116, 117].

Inhibition of A2AR, such as with inupadenant, enhances tumor vaccine efficacy and synergizes with ICIs [118]. In murine T cell lymphoma models, A2AR inhibition reduces tumor growth and boosts IFN-γ levels [119]. Preclinical investigations have also indicated favorable pharmacokinetic attributes associated with A2AR blockade [120].

The potential therapeutic value of A2AR is underscored by its significance in certain cohorts, such as the male Chinese Glioma Genome Atlas and Moroccan glioma patients [121]. Nevertheless, it is essential to acknowledge that a subset of patients may face challenges in restoring immune responses [117]. Studies employing A2AR antagonists or genetic knockout of A2AR in immune cells have unequivocally demonstrated that interception of the adenosine-A2AR signaling pathway markedly enhances antitumor immune responses [117].

A2AR blockade in cancer immunotherapy warrants further clinical investigation, both alone and in combination with other immunotherapeutic modalities.

#### B7-H3

In gliomas, B7-H3 (CD276), an immune checkpoint, modulates the TME and T-cell function. Its overexpression correlates with advanced disease stages and poorer prognoses, highlighting its significance in glioma pathogenesis. B7-H3 interacts with receptors (TLT-2, IL20RA, PLA2R1, etc.) influencing immune responses, often promoting tumor immunosuppression. Its interaction with TLT-2 on immune cells, including CD8+ T cells, has contradictory effects, supporting proinflammatory responses or reducing Th1 immune activity. It is expressed in hematological cancers and solid tumors, including high-grade gliomas [122]. B7-H3 inhibits T-cell activation and proliferation, driving tumor immune evasion via diverse signaling pathways [123]. Aberrant upregulation across cancers, especially in more than half of glioblastoma cases, makes it a promising therapeutic target [124].

CAR-T cell therapy targeting B7-H3 shows promise in preclinical studies. A phase I/II clinical trial initiated in 2022 assessed B7-H3 CAR-T-cell therapy in recurrent/refractory glioblastoma [125]. Clinical trials of drugs like MGC018, TRX518, and ITC-6102RO are also underway. [122].

#### VISTA

In the context of gliomas, VISTA is predominantly expressed in T cells and TAMs, correlating with overall survival and glioma grade. This expression pattern reveals VISTA's significant role in the immunosuppressive landscape of gliomas [126]. Additionally, VISTA's regulation of monocyte migration and activation, primarily through the CCL2/CCR2 axis, underscores its critical function in cancer development and metastasis. Furthermore, MDSCs upregulate VISTA under hypoxic conditions, highlighting HIF1α as a key transcriptional activator [127].

VISTA is highly expressed in gliomas and is positively correlated with critical immune checkpoints like PD-1, correlating with an unfavorable prognosis [128]. Preclinical models have demonstrated VISTA's involvement in restraining antitumor T-cell responses, thereby fostering tumor progression, while its blockade augments antitumor immune responses, culminating in enhanced survival in murine models of melanoma, colon cancer, and lung cancer [129, 130]. Further comprehensive investigations are needed to clarify VISTA's regulatory role in anticancer immunity [130].

Presently, ongoing clinical trials are evaluating a monoclonal antibody and a small molecule specifically targeting VISTA [131]. Furthermore, VISTA has emerged as a promising focal point for combination therapy alongside other ICIs, including anti-PD-1 and anti-CTLA-4 agents [129]. VISTA is a promising target in cancer immunotherapy, and warrants further investigation [131, 132].

#### B7-H4

The expression of B7-H4 in TME is regulated by cytokines such as IL6 and IL10, with IL6-activated STAT3 enhancing its expression [133]. B7-H4's presence, particularly on macrophages/microglia, constitutes a significant immunosuppressive mechanism, hindering effective T-cell responses and leading to tumor progression.

Expressed on TAMs in various malignancies, including colorectal, pancreatic, ovarian, breast, and renal cancers, B7-H4 is clinically significant due to its association with adverse indicators of tumor aggressiveness [134–136]. Mechanistically, it suppresses inflammatory CD4+ T-cell responses and links TAMs expressing B7-H4 with FoxP3+ Tregs in the TME [134]. Targeting B7-H4 in diverse cancer types holds promise for reshaping the TME for antigen-specific tumor cell elimination [134].

In preclinical studies, B7-H4 blockade enhances T cell immune responses in bladder urothelial carcinoma patients and shows potential in combination with PD-L1 blockade in breast cancer, intensifying the anti-tumor immune response [137]. However, a comprehensive understanding of B7-H4's role in cancer immunity requires further exploration, as its regulatory influence remains unknown [134].

Preliminary findings suggest B7-H4 as a potential treatment for colorectal cancer [136]. Consequently, further research is essential to determine the clinical effectiveness of B7-H4 blockade for glioma treatment, either as a monotherapy or in combination with other immunotherapies.

#### SIRPα

Signal-regulatory protein alpha (SIRPα) is an immune checkpoint protein. Its presence is noted on both tumor cells and TAMs across a spectrum of cancers, including melanoma, esophageal squamous cell carcinoma, and spinal chordoma [138–140]. However, discerning SIRPα's intricate role in cancer immunity presents a challenge, and its precise regulatory function in anticancer immunity requires further elucidation [139, 141].

Preclinical models have revealed a dual role for SIRPα: while it enhances antitumor immunity, it also functions as a pivotal inhibitory immune modulator in macrophages. Notably, the absence of SIRPα expression signifies melanoma dedifferentiation, a critical phenotype associated with immunotherapy efficacy. Inhibiting SIRPα in melanoma cells hinders tumor eradication by activated CD8+ T cells within a coculture setup. Mice harboring SIRPα-deficient melanoma tumors exhibit no response to anti-PD-L1 treatment, underscoring the significant impact of melanoma-specific SIRPα overexpression on immunotherapy response. Mechanistically, SIRPα regulation is mediated by its pseudogene, SIRPΑP1 [139]. In esophageal squamous cell carcinoma, elevated SIRPα expression correlates positively with a poorer prognosis, potentially by impeding macrophage-mediated phagocytosis of tumor cells and fostering an immunosuppressive microenvironment [140]. In the context of spinal chordoma, targeting the CD47/SIRPα signaling pathway has demonstrated efficacy in augmenting macrophage phagocytosis and facilitating immune evasion [138].

Despite the progress made with *SIRPα* in cancer research, its benefits have not yet been applied to the field of glioma. Presently, clinical trials involving the anti-SIRPα monoclonal antibody BI 770371, evorpacept, and the SIRPα-4-1BBL fusion protein DSP107, among many others, are underway across various other cancer types [142, 143]. However, comprehensive studies are warranted to ascertain the clinical effectiveness of SIRPα blockade, either as a standalone therapy or in conjunction with other immunotherapies in glioma [144].

#### LILRB2

Leukocyte immunoglobulin-like receptor subfamily B member 2 (LILRB2), an immune checkpoint protein expressed on myeloid cells, exhibits involvement in GBM progression [145, 146]. Research by Oushy *et al.* reveals that LILRB2-containing small extracellular vesicles originating from GBM cells foster tumor advancement by stimulating the generation and proliferation of MDSCs [146]. As known suppressors of the immune system, MDSCs' induction within the TME correlates with adverse prognosis in GBM patients [146].

Moreover, Li *et al.*'s study indicates that Siglecs, including LILRB2, represent promising targets for immunotherapy, potentially augmenting the efficacy of current ICIs in glioma immunotherapy [147]. Additionally, Zhang *et al.* demonstrated that blocking LILRB2 induces reprogramming of tumor-associated myeloid cells, instigating an antitumor immune response [148]. This suggests that LILRB2 may function as a myeloid immune checkpoint, reshaping the tumor-associated myeloid landscape and eliciting anti-tumor immunity. Thus, the ongoing exploration of LILRB2 and other immune checkpoints in this context holds promise for the development of novel immunotherapeutic strategies benefiting GBM patients [149].

### Natural killer cell- related immunoregulatory molecules

In GBM, NK cells, identified by the CD56 marker, are present in perivascular tumor regions but are often functionally impaired in the peripheral blood of GBM patients [150]. This impairment influences their capability for tumor cell lysis, which is also modulated by surface molecules on glioblastoma stem-like cells (GSLCs), including CD44, CD54, MHC class I, and PD-L1​.

The development of chimeric antigen receptor (CAR)-NK cell therapies has significantly advanced the field. By genetically modifying NK cells to express CARs targeting specific glioma antigens, these therapies enhance the specificity and efficacy of NK cell-mediated tumor destruction [151]. CAR-NK cells offer several advantages over traditional CAR-T therapies, including the potential for "off-the-shelf" availability and reduced risk of graft versus host disease, making them particularly attractive for solid tumors like gliomas [151].

#### KIRs

Killer cell immunoglobulin-like receptors (KIRs) on NK cells play a critical role in NK cell function by interacting with MHC class I molecules on target cells. In glioma, increased KIR expression on NK cells and decreased ligand expression on tumor cells reduce NK cell activity [152]. However, studies indicate that activating KIR genes enhances NK cell efficacy against pediatric tumors [153]. Alterations in the glioblastoma microenvironment, induced by oncolytic virotherapy or combined immunotherapy with radiotherapy, lead to heightened KIR expression on NK cells, making KIRs a potential target for glioma immunotherapy [154, 155]. These observations posit KIRs as plausible targets for glioma immunotherapy. But notably, inhibiting inhibitory KIR receptors may not yield favorable outcomes, as KIR-negative NK cells exhibit a mature but hypo-responsive phenotype, reducing their cytotoxicity compared to that of wild-type NK cells [153].

#### NKG2A

Natural killer group 2A (NKG2A), a checkpoint receptor on NK and CD8+ T cells, pairs with CD94 to form a heterodimer targeting HLA-E molecules, thus inhibiting NK and CD8+ T-cell activity [156]. NKG2A unblocking enhances cytotoxic lymphocyte functions, presenting a promising avenue for countering tumor immune evasion and developing antitumor therapies [156]. Inhibition of NKG2A bolsters tumor immunity and NK/CD8+ T-cell effector functions, as shown in vitro and in vivo studies [157].

NKG2A inhibitor Monalizumab, currently in phase 2 trials, has demonstrated limited efficacy in head and neck squamous cell carcinoma (HNSCC). NKG2A blockade also amplifies CD8+ T cell response to cancer vaccines, underscoring its potential in immunotherapy [157].

The antibody-mediated blockade of NKG2A has shown an acceptable safety profile in clinical trials, often combined with other therapeutic antibodies. In tumor immunotherapy, combining agents is key, positioning NKG2A as an ideal candidate for such strategies due to its regulatory role in both adaptive and innate immunity.

To optimize NK cell-based therapies for gliomas, combining them with other treatments, such as chemotherapy, is crucial. This combination can increase the susceptibility of GSLCs to NK cell-mediated lysis by altering the expression of key surface molecules. Additionally, understanding and manipulating the receptor-ligand interactions between NK cells and glioma cells are vital for improving the effectiveness of these therapies.

### Other immunoregulatory molecules

#### HVEM

Herpesvirus entry mediator (HVEM), of the TNFR superfamily, regulates immune responses and is ubiquitously present in T cells, B cells, and NK cells. It interacts with multiple ligands, including LIGHT (TNFSF14) and B and T lymphocyte attenuator (BTLA) [158, 159]. The HVEM-BTLA axis inhibits T-cell activation and proliferation, while HVEM-LIGHT enhances these processes [158, 160]. HVEM's role in tumor development and immune evasion, particularly in glioblastoma, underscores its therapeutic potential for cancer treatment [158, 161].

The HVEM-BTLA interaction is critical in maintaining immune balance, dampening T-cell activation, and fostering immune tolerance [162, 163]. However, in glioblastoma, this interaction contributes to tumor progression by impairing T-cell-mediated immunity, highlighting the therapeutic value of disrupting this pathway [161].

Emerging strategies to inhibit HVEM-BTLA interaction include monoclonal antibodies and soluble HVEM. For instance, the anti-BTLA monoclonal antibody HFB200603 has shown efficacy in disrupting this interaction, enhancing antitumor immunity in melanoma and NSCLC [164]. Additionally, an HVEM-Fc fusion protein has proven effective in blocking this interaction and boosting T-cell responses in experimental settings, suggesting a promising avenue for enhancing solid tumor therapies.

#### HLA-G

HLA-G, a nonclassical MHC component, plays a crucial role in immune tolerance and is expressed in various immune and tumor cells. Its heightened presence in cancers, such as glioblastoma, is associated with immune evasion and poor prognosis [165, 166]. HLA-G interacts with receptors like LILRB1, LILRB2, and KIR2DL4 to inhibit immune responses, highlighting its potential as an immune checkpoint target [166]. Strategies to counteract HLA-G, including monoclonal antibodies and small molecule inhibitors, have shown promise in preclinical studies. The anti-HLA-G monoclonal antibody 87G, for example, counters immune suppression and enhances anti-tumor immunity in vitro and in vivo [167]. Similarly, the small molecule inhibitor 4-iodo-6-phenylpyrimidine reduced HLA-G expression and increased T-cell mediated responses in laboratory and animal models [165]. However, the clinical efficacy of these approaches remains to be validated.

#### IDO-1

Indoleamine 2,3-dioxygenase 1 (IDO-1), an enzyme critical in tryptophan-to-kynurenine conversion, plays a pivotal role in modulating T-cell mediated immunity via aryl hydrocarbon receptor activation, a process integral to immune evasion in cancers such as glioblastoma, and correlates with poor prognosis [152, 168, 169]. The emergence of IDO-1 inhibitors, including small molecules and monoclonal antibodies, has led to significant progress in cancer therapy, with several drugs advancing in clinical trials [170, 171].

Among these, epacadostat, a small molecule inhibitor, demonstrated limited efficacy in trials, both as monotherapy and in combination treatments [169]. Contrastingly, BMS-986205 and Indoximod show notable promise. BMS-986205, a selective inhibitor, uniquely targets IDO-1, differentiating it from IDO-2, and has shown efficacy in preclinical trials by inhibiting IDO-1 and enhancing T-cell responses [172]. Indoximod, granted US FDA orphan-drug status for melanoma, not only impedes IDO-1 activity but also mimics tryptophan, countering the inhibition of the mammalian target of rapamycin complex 1, as recent studies reveal [172].

#### CD40

The interplay between CD40 and the glioma microenvironment reveals a nuanced and multifaceted role of this TNF receptor family member in glioma biology. The regulation of vascular endothelial growth factor (VEGF) by CD40 underscores its pivotal role in modulating tumor angiogenesis. [173, 174]. Agonistic CD40 therapies, notably αCD40-induced tertiary lymphoid structures, are instrumental in shaping the immune landscape within gliomas. These structures, comprised of diverse immune cells including T cells, B cells, macrophages, and DCs, reflect CD40's influence on immune cell functionality, particularly T-cell activity [173, 175]. However, this therapeutic approach also presents a paradox, as it has been shown to impede responses to checkpoint blockade in gliomas, indicating a complex interplay between enhancing anti-tumor immunity and potentially limiting therapeutic efficacy.

Agonistic CD40 antibodies (αCD40), such as selicrelumab, have shown potential in cancer treatment, exhibiting positive results in both preclinical and clinical scenarios. These antibodies reorient macrophages towards an anti-tumor phenotype and enhance DC-mediated antigen presentation. Additionally, CD40 activation plays a critical role in B-cell functions including activation, antibody production, germinal center formation, and antigen presentation. Various solid tumors are currently being targeted in clinical trials involving αCD40 antibodies [176].

As mentioned above, recent studies also indicate that αCD40 therapy, while promoting tertiary lymphoid structures, can attenuate the response to checkpoint blockade in gliomas [173]. Gliomas are characterized by a high presence of bone marrow-derived macrophages and microglia, which contribute to tumor growth and immune evasion. Post-treatment, αCD40 therapy leads to an increase in systemic suppressive CD11b+ B cells and a shift in the TME. Therefore, a combinatorial approach with checkpoint inhibitors might be necessary to enhance the therapeutic efficacy of αCD40.

#### NOX2

Nicotinamide adenine dinucleotide phosphate hydrogen oxidase 2 (NOX2) plays a pivotal role in redox signaling by facilitating reactive oxygen species production. Its overexpression in GBM correlates with mesenchymal phenotype development in glioma cells and is linked to diminished survival outcomes [177]. NOX2 suppression offers a potential therapeutic strategy against hypoxia-driven tumor progression in GBM [178].

However, the exact role of NOX2 in tumor dynamics has not been fully elucidated, and existing NOX inhibitors lack targeted specificity [179]. Therefore, further research into NOX2 modulation as a means to augment lymphocyte-mediated antimetastatic response is imperative [180].

## Conclusions

GBM's intratumoral heterogeneity, a crux of its resistance to broad-spectrum immunotherapies, necessitates a granular understanding of how different tumor subclones manipulate the immune microenvironment. Concurrently, the TME of GBM, characterized by its immunosuppressive nature, requires strategies that transcend mere immune activation. This process includes remodeling the TME to foster immune infiltration and response. Furthermore, the potential synergy between immunotherapy and other modalities, like targeted radiation, opens avenues for combinatorial therapies that could potentiate immune responses while mitigating toxicity. A pivotal yet underexplored frontier is the development of personalized immunotherapies guided by genomic and proteomic profiling that target unique mutations and pathways in individual tumors. Additionally, the establishment of long-term immune memory against GBM recurrence remains an elusive goal. Investigating vaccines and methods to bolster durable immune surveillance could redefine therapeutic approaches. These approaches could improve patient outcomes, signaling a new era in the fight against gliomas.

### Supplementary Information

**Additional file 1: Supplementary Table S1.** Overview of ICIs in clinical trials for non-glioma cancer immunotherapies.

## Data Availability

Not applicable.

## Abbreviations

PD-1: Programmed Death-1
VISTA: V-domain Ig Suppressor of T cell Activation
NKG2A: Natural Killer Group 2, Member A
ICOS: Inducible T-cell Co-stimulator
A2AR: Adenosine A2A Receptor
LAG-3: Lymphocyte-activation Gene 3
IDO: Indoleamine 2,3-Dioxygenase
PD-L1: Programmed Death-Ligand 1
GITR: Glucocorticoid-Induced TNFR-Related Protein
OX40: A Member of the TNF-Receptor Superfamily
CD40: A TNF Receptor Family Member
NOX2: Nicotinamide Adenine Dinucleotide Phosphate Hydrogen Oxidase 2
SIRPα: Signal-regulatory Protein Alpha
LILRB2: Leukocyte Immunoglobulin-like Receptor Subfamily B Member 2
BTLA: B and T Lymphocyte Attenuator
LIGHT: A Ligand Interacting with HVEM
TAM: Tumor-Associated Macrophage
MDSC: Myeloid-Derived Suppressor Cell
ICI: Immune Checkpoint Inhibitor
NSCLC: Non-Small Cell Lung Cancer
RCC: Renal Cell Carcinoma
GBM: Glioblastoma Multiforme
TME: Tumor Microenvironment
TCR: T Cell Receptor
APC: Antigen-Presenting Cell
Treg: Regulatory T cell
GAM: Glioma-Associated Macrophage
TIL: Tumor-Infiltrating Lymphocyte
IDH: Isocitrate Dehydrogenase
TIM-3: T-cell immunoglobulin and Mucin-Domain Containing-3
DC: Dendritic Cell
TIGIT: T Cell Immunoreceptor with Ig and ITIM Domains
GSLC: Glioblastoma Stem-Like Cell
KIR: Killer Cell Immunoglobulin-Like Receptor
NKG2A: Natural Killer Group 2A
HVEM: Herpesvirus Entry Mediator
e.g.: *Exempli gratia* (Example)
Etc.: *Et cetera*
