# Supplementary material for: From glioma gloom to immune bloom: unveiling novel immunotherapeutic paradigms-a review
Source: J Exp Clin Cancer Res. 2024 Feb 12;43:47. doi: 10.1186/s13046-024-02973-5 (PMC10860318; doi:10.1186/s13046-024-02973-5)
Supplement: Supplementary file 1 — Additional file 1: Supplementary Table S1. Overview of ICIs in clinical trials for non-glioma cancer immunotherapies. [file 13046_2024_2973_MOESM1_ESM.docx]

| Target | Intervention | Author | Year | Journal | Country | Clinical Trial Phase | **Enrolment** | Comments |
| --- | --- | --- | --- | --- | --- | --- | --- | --- |
| PD-1 | Pembrolizumab (Keytruda)  Nivolumab (Opdivo)  Cemiplimab (Libtayo)  Dostarlimab  HX009^a^ | Reck *et al.*  Schmid *et al.*  Topalian *et al.*  Gross *et al.*  Ferrarotto *et al.*  Rischin *et al.*  Yap *et al.*  -  -  - | 2016  2022  2019  2022  2021  2021  2022  -  -  - | *N Engl J Med.*  *N Engl J Med.*  *JAMA Oncol.*  *N Engl J Med.*  *Clin Cancer Res.*  *J Immunother Cancer.*  *J Immunother Cancer.*  *-*  *-*  *-* | N.A.  US  US  US  US  Multinational  US  AU  CN  CN | III  III  I  II  II  II  Ib  I  I  I | 305  1174  395  79  40  432  58  21  25  210 | Median progression-free survival was 10.3 months in the pembrolizumab group versus 6.0 months in the chemotherapy group.  Estimated event-free survival at 36 months was 84.5% in the pembrolizumab-chemotherapy group, as compared with 76.8% in the placebo-chemotherapy group.  Nivolumab treatment was associated with long-term survival in a subset of heavily pretreated patients with advanced melanoma, renal cell carcinoma, or NSCLC.  Pathological complete response was observed in 51% of patients and a pathological major response in 13% of the patients.  Cemiplimab immunotherapy in locoregionally advanced, resectable cutaneous HNSCC was safe and induced a high pathologic response rate.  Substantial clinical activity of cemiplimab in cutaneous squamous cell carcinoma patients was confirmed.  Dostarlimab was well tolerated in both doublet and triplet regimens tested, with promising antitumor activity observed with all combinations.  Unpublished: ClinicalTrials.gov ID NCT04097769  Unpublished: ClinicalTrials.gov ID NCT05731752  Unpublished: ClinicalTrials.gov ID NCT04886271 |
| PD-L1 | Atezolizumab  Avelumab  Durvalumab  Spartalizumab  PF-07257876^a^  IBI322^a^ | Finn *et al.*  Herbst *et al.*  Liu *et al.*  Powless *et al.*  Spigel *et al.*  Johnson *et al.*  Schöffski *et al.* | 2020  2020  2021  2020  2022  2023  2022 | *N Eng J Med.*  *N Eng J Med.*  *J Clin Oncol.*  *N Eng J Med.*  *J Clin Oncol.*  *J Clin Oncol.*  *J Immunother Cancer.* | Multinational  Multinational  Multinational  Multinational  Multinational  Multinational  Multinational | III  III  I/III  III  III  III  I/II | 558  572  503  700  713  1186  490 | In patients with unresectable hepatocellular carcinoma, atezolizumab combined with bevacizumab resulted in better overall and progression-free survival outcomes than kinase inhibitor sorafenib.  Atezolizumab treatment resulted in significantly longer overall survival than platinum-based chemotherapy among patients with NSCLC, regardless of histologic type.  Adding atezolizumab as a treatment for extensive-stage small-cell lung cancer continued to demonstrate improved overall survival and a tolerable safety profile.  Maintenance of avelumab plus best supportive care significantly prolonged overall survival, as compared with best supportive care alone, among patients with urothelial cancer who had disease that had not progressed with first-line chemotherapy.  Sustained overall survival and progression-free survival were observed with durvalumab after chemoradiotherapy.  Progression-free survival was significantly improved with durvalumab + chemotherapy versus chemotherapy alone.  Spartalizumab was well tolerated in combination with ieramilimab. |
| CTLA-4 | Ipilimumab  Tremelimumab | Sun *et al.*  Slovin *et al.*  Hersh *et al.*  Siu *et al.*  Baverel *et al.*  Subudhi *et al.* | 2021  2013  2011  2019  2019  2021 | *Prostate.*  *Ann Oncol*  *Invest New Drugs.*  *JAMA Oncol.*  *Clin Transl Sci.*  *J Immunother Cancer.* | US  US  US  Multinational  Multinational  US | I/II  I/II  II  II  II  II | 75  75  72  267  571  31 | Demonstrated potential in treating metastatic castrate-resistant prostate cancer, suggesting application in a wider range of prostate cancer patients.  In metastatic castrate-resistant prostate cancer patients, ipilimumab 10 mg/kg ± radiotherapy suggested clinical antitumor activity with disease control and manageable adverse effects.  Ipilimumab therapy resulted in clinically meaningful responses in advanced melanoma patients.  Tremelimumab exhibited a manageable toxicity profile in patients with recurrent or metastatic HNSCC and low or no PD-L1 tumor cell expression,  Tremelimumab demonstrated no clinically meaningful differences in overall survival in patients with unresectable malignant mesothelioma.  Tremelimumab was safe and well tolerated in patients with chemotherapy-naive metastatic castrate-resistant prostate cancer to bone, with potential activity in a small number of patients as measured by radiographic progression-free survival |
| TIM-3 | Sym023  Spartalizumab  TSR-022 | -  -  -  Curigliano *et al.*  -  -  - | -  -  -  2021  -  -  - | *-*  *-*  *-*  *Clin Cancer Res.*  *-*  *-*  *-* | US  Multinational  US  Multinational  US  US, ES  US | I  I  I  I-Ib/II  II  I  II | 24  148  89  252  42  475  56 | Unpublished: ClinicalTrials.gov ID NCT03489343  Unpublished: ClinicalTrials.gov ID NCT04641871  Unpublished: ClinicalTrials.gov ID NCT03311412  Sabatolimab was well tolerated and showed preliminary signs of antitumor activity.  Unpublished: ClinicalTrials.gov ID NCT03680508  Unpublished: ClinicalTrials.gov ID NCT02817633  Unpublished: ClinicalTrials.gov ID NCT04139902 |
| TIGIT | Tiragolumab  Etigilimab  Vibostolimab | Cho *et al.*  Mettu *et al.*  Niu *et al.* | 2022  2022  2022 | *Lancet Oncol.*  *Clin Cancer Res.*  *Ann Oncol.* | Multinational  US  Multinational | II  Ia/b  I | 135  33  492 | Tiragolumab showed a clinically meaningful improvement in objective response rate and progression-free survival in patients with chemotherapy-naive, PD-L1-positive, recurrent, or metastatic NSCLC.  Etigilimab had an acceptable safety profile with preliminary evidence of clinical benefits.  Vibostolimab was well tolerated and demonstrated antitumor activity in patients with advanced solid tumors, including patients with advanced NSCLC. |
| VISTA | CA-170  CI-8993  JNJ-61610588 | -  -  - | -  -  - | *-*  *-*  *-* | Multinational  US, AU  US | I  I  I | 71  26  12 | Unpublished: ClinicalTrials.gov ID NCT02812875  Unpublished: ClinicalTrials.gov ID NCT04475523  Unpublished: ClinicalTrials.gov ID NCT02671955 |
| NKG2A | Monalizumab | Galot *et al.* | 2021 | *Eur J Cancer.* | EU | II | 340 | Monalizumab monotherapy showed limited activity in recurrent or metastatic HNSCCs. |
| ICOS | Vopratelimab^a^  Feladilimab^a^ | Yap *et al.*  Hansen *et al.* | 2022  2020 | *Clin Cancer Res.*  *J Clin Oncol.* | US  Multinational | I/II  II/III | 242  315 | Vopratelimab was well tolerated in patients with advanced solid tumors but did not confer durable antitumor activity.  Result unpublished: ClinicalTrials.gov ID NCT04128696 |
| SIRPα | FSI-0189  BR105  BI765063, 754091  SL-172154^a^  TTI-622  Evorpacept  BI 770371  DSP107 | Narkhede *et al.*  -  -  -  -  -  Lakhani *et al.*  *-*  Cendrowicz *et al.* | 2023  -  -  -  -  -  2021  -  2022 | *EJHaem.*  *-*  *-*  *-*  *-*  *-*  *Lancet Oncol.*  *-*  *J Exp Clin Cancer Res.* | US  CN  FR  BE  JP  US  US, KR  US  - | I  I  I  I  I  I  I  I  - | 9  162  116  18  34  32  174  42  - | Result unpublished: ClinicalTrials.gov ID NCT04502706  Unpublished: ClinicalTrials.gov ID NCT05351697  Unpublished: ClinicalTrials.gov ID NCT03990233  Unpublished: ClinicalTrials.gov ID NCT04653142  Unpublished: ClinicalTrials.gov ID NCT04406623  Unpublished: ClinicalTrials.gov ID NCT05139225  Safety findings supported the use of evorpacept for patients with advanced solid tumors, and preliminary antitumor activity results supported future investigation of evorpacept use in patients with HNSCC, gastric or gastroesophageal junction cancer, and NSCLC.  Unpublished: ClinicalTrials.gov ID NCT05327946  DSP107 (re)activated innate and adaptive anticancer immune responses and was found to be of therapeutic use alone and in combination with rituximab for the treatment of diffuse large B-cell lymphoma patients. |
| CD47 | Letaplimab (IBI188)  Magrolimab  ZL-1201  STI-6643  CC-9002  AK117  A0-176  IMC-002  TQB2928  HX009^a^  PF-07257876^a^  CPO107(JMP601)^a^  IBI322^a^  TG-1801^a^  IMM0306^a^ | Ni *et al.*  Paul *et al.*  -  -  Narla *et al.*  -  -  Andrejeva *et al.*  -  -  -  -  -  -  -  Chauchet *et al.*  - | 2021  2023  -  -  2022  -  2021  -  -  -  -  -  -  -  2022  - | *Cancer Immunol Immunother.*  *Future Oncol.*  *-*  *-*  *Cancer Immunol Immunother.*  *-*  *-*  *J Immunol.*  *-*  *-*  *-*  *-*  *-*  *-*  *-*  *Exp Hematol Oncol.*  *-* | CN  CZ, US, CA  US, CN US US, ES  CN CN US US, KR N.A  US, ES US CN US CN US  US | Ia  II  I  I  I/II  I  I/II  I/II  I  I  I  I  I  Ia  Ia/Ib  Ib  I | 49  153  66  24  60  162  190  132  24  20  90  75  182  45  218  60  131 | IBI188 treatment increased vascular endothelial growth factor A (VEGF-A) levels in a solid tumor model, and combined treatment with an anti-VEGF-A antibody resulted in an enhanced antitumor effect.  Result Unpublished: ClinicalTrials.gov ID NCT04892446  Unpublished: ClinicalTrials.gov ID NCT04257617  Unpublished: ClinicalTrials.gov ID NCT04900519  CC-90002 displayed acceptable pharmacokinetic properties and a favorable toxicity profile.  Unpublished: ClinicalTrials.gov ID NCT04728334  Unpublished: ClinicalTrials.gov ID NCT04900350  SIRPα combined with AO-176 enhanced phagocytosis  Unpublished: ClinicalTrials.gov ID NCT04306224  Unpublished: ClinicalTrials.gov ID NCT04854681  Unpublished: ClinicalTrials.gov ID NCT04881045  Unpublished: ClinicalTrials.gov ID NCT04853329  Unpublished: ClinicalTrials.gov ID NCT04795128  Unpublished: ClinicalTrials.gov ID NCT04338659  Unpublished: ClinicalTrials.gov ID NCT04328831  NI-1701 treatment transformed the tumor microenvironment into a more antitumorigenic state with increased NK cells, monocytes, DC and MHC-II tumor-associated macrophages, and decreased granulocytic MDSCs.  Unpublished: ClinicalTrials.gov ID NCT04746131 |
| LILRB2 | IO-108 | - | - | *-* | US | Ib | 309 | Unpublished: ClinicalTrials.gov ID NCT05054348 |
| LILRB4 | IO-202 | - | - | *-* | US | I | 122 | Unpublished: ClinicalTrials.gov ID NCT04372433 |
| BTLA | HFB200603 | - | - | *-* | US, ES | Ia/b | 83 | Unpublished: ClinicalTrials.gov ID NCT05789069 |
| IDO1/2 | Indoximod  SHR9146  KHK2455  IO102  Epacadostat  BMS-986205 | Johnson *et al.*  -  -  -  Kjeldson *et al.*  *-*  *-* | 2023  -  -  -  2021  -  - | *Neuro Oncol.*  *-*  *-*  *-*  *Nat Med.*  *-*  *-* | US  CN  US, FR  US, ES  DK  US  US | I  I  I  I  I/II  I  II | 81  23  36  16  50  15  24 | Indoximod was well tolerated and could be safely combined with chemotherapy and radiation  Unpublished: ClinicalTrials.gov ID NCT03491631  Unpublished: ClinicalTrials.gov ID NCT02867007  Unpublished: ClinicalTrials.gov ID NCT03915405  Immune-modulating IDO/PD-L1-targeting vaccine combined with nivolumab achieved promising clinical activity and long-lasting immune responses in patients with advanced melanoma.  Unpublished: ClinicalTrials.gov ID NCT03217669  Unpublished: ClinicalTrials.gov ID NCT04106414 |
| GITR | TRX518  BMS-986156  Vopratelimab^a^  Feladilimab^a^ | Davar *et al.*  *Heinhuis et al.* | 2022  2020 | *Clin Cancer Res.*  *JAMA Oncol.* | US  Multinational | I  I/II | 109  295 | Repeated dose TRX518 monotherapy and in combination resulted in limited clinical responses associated with immune activation.  BMS-986156 was found to have a manageable safety profile |
| OX40 | MEDI6383  GSK3174998  MOXR0916 | -  Vinay *et al.*  Kim *et al.* | -  2023  2022 | *-*  *J Immunother Cancer.*  *Clin Cancer Res.* | US, AU  US, FR, CA, NL  Multinational | I  I  I | 39  141  174 | Unpublished: ClinicalTrials.gov ID NCT02221960  GSK3174998 was well tolerated over the dose range tested and demonstrated target engagement.  The favorable safety profile and evidence of tumor immune activation in a subset of patients supported further investigation. |
| 4-1BB | Urelumab  Utomilumab | Timmerman *et al.*  Hamid *et al.*  Hong *et al.* | 2020  2022  2022 | *Am J Hematol.*  *J Immunother Cancer.*  *Front Immunol.* | US, ES, FR  US, JP, FR, NL  Multinational | I  I  I | 124  174  190 | Showed manageable safety in B‐cell lymphoma, but did not enhance clinical activity.  Utomilumab was well tolerated and demonstrated preliminary antitumor activity in selected groups of patients.  Utomilumab was well tolerated, but antitumor activity was low in patients. |
| CD27 | Varlilumab | Sanborn *et al.*  Burris *et al.*  Ansell *et al.* | 2022  2017  2020 | *J Immunother Cancer.*  *J Clin Oncol.*  *Blood Adv.* | US  US  US | I/II  I  I | 175  90  90 | Varlilumab was well tolerated, without significant toxicity  Varlilumab used 10 mg/kg was well tolerated. Varlilumab was biologically and clinically active.  Supports further investigation of varlilumab for hematologic malignancies, particularly in combination approaches targeting nonredundant immune regulating pathways. |
| CD28 | Abatacept  Belatacept | Westhovens *et al.*  -  Vincenti *et al.* | 2014  -  2016 | *Clin Exp Rheumatol.*  *-*  *N Engl J Med.* | US  N.A  Worldwide | IIb  III  III | 524  738  738 | Abatacept in combination with methotrexate demonstrated consistent safety and sustained efficacy.  Unpublished: ClinicalTrials.gov ID NCT00048581  Long-term patient and graft survival after organ transplant were significantly higher with belatacept use. |
| CD40 | Selicrelumab | Byrne *et al.*  Machiels *et al.* | 2021  2020 | *Clin Cancer Res.*  *J Immunother Cancer.* | US  US, FR, BE | I  I | 19  38 | Selicrelumab induced changes in the tumor microenvironment in patients with resectable pancreatic cancer.  Selicrelumab in a combination with emactuzumab demonstrated a manageable safety profile and evidence of PD activity but did not translate into objective clinical responses. |
| B7-H3 | MGC018/9  TRX518  B7-H3 CAR-T | -  -  *-* | -  -  - | *-*  *-*  *-* | US  US  US  CN | I/Ib  I  I  I/II | 278  109  10  40 | Unpublished: ClinicalTrials.gov ID NCT05293496  Unpublished: ClinicalTrials.gov ID NCT02628574  Unpublished: ClinicalTrials.gov ID NCT01239134  Unpublished: ClinicalTrials.gov ID NCT04077866 |
| A2AR | Inupadenant | -  -  - | -  -  - | *-*  *-*  *-* | Multinational  UK, BE  US, UK, BE | I/Ib  I  II | 119  64  192 | Unpublished: ClinicalTrials.gov ID NCT03873883  Unpublished: ClinicalTrials.gov ID NCT05117177  Unpublished: ClinicalTrials.gov ID NCT05403385 |
| LAG-3 | Relatlimab  Eftilagimod alpha  (IMP321)  Flianlimab | Tawbi *et al.*  Amaria *et al.*  Brignone *et al.*  *-* | 2022  2022  2009  - | *N Eng J Med.*  *Nature.*  *Clin Cancer Res.*  *-* | Multinational  US  FR  AU, US | II/III  II  I  II/III | 714  53  24  850 | The use of relatlimab and nivolumab together provided a greater benefit with regard to progression-free survival than inhibition of PD-1 alone in patients with previously untreated metastatic or unresectable melanoma.  Neoadjuvant relatlimab and nivolumab induced a high pathologic complete response rate.  IMP321 was found to inhibit tumor growth and prolong the progression-free survival of advanced RCC patients.  Unpublished: ClinicalTrials.gov ID NCT05785767 |
| CD40L | SL-172154^a^ |  | | | | | | |
| CD19 | TG-1801^a^ |  |  |  |  |  |  |  |
| CD20 | IMM0306^a^  CPO107(JMP601)^a^ |  |  |  |  |  |  |  |

a: Bispecific antibodies

N.A.: Not available
